# Molecular Cloning and Functional Characterization of a Novel Isoflavone 3′-*O-*methyltransferase from *Pueraria lobata*

**DOI:** 10.3389/fpls.2016.00793

**Published:** 2016-06-06

**Authors:** Jia Li, Changfu Li, Junbo Gou, Yansheng Zhang

**Affiliations:** ^1^CAS Key Laboratory of Plant Germplasm Enhancement and Specialty Agriculture, Wuhan Botanical Garden, Chinese Academy of SciencesWuhan, China; ^2^University of Chinese Academy of SciencesBeijing, China

**Keywords:** methyltransferase, methyl jasmonate, isoflavone, 3′-*O*-methylation, *Pueraria lobata*

## Abstract

*Pueraria lobata* roots accumulate 3′-, 4′- and 7-*O*-methylated isoflavones and many of these methylated compounds exhibit various pharmacological activities. Either the 4′- or 7-*O*-methylation activity has been investigated at molecular levels in several legume species. However, the gene encoding the isoflavone 3′-*O-*methyltransferase (OMT) has not yet been isolated from any plant species. In this study, we reported the first cDNA encoding the isoflavone 3′-OMT from *P. lobata* (designated PlOMT4). Heterologous expressions in yeast and *Escherichia coli* cells showed that the gene product exhibits an enzyme activity to methylate the 3′-hydroxy group of the isoflavone substrate. The transcript abundance of *PlOMT4* matches well with its enzymatic product in different organs of *P. lobata* and in the plant roots in response to methyl jasmonate elicitation. Integration of the biochemical with metabolic and transcript data supported the proposed function of PlOMT4. The identification of *PlOMT4* would not only help to understand the isoflavonoid metabolism in *P. lobata* but also potentially provide an enzyme catalyst for methylating existing drug candidates to improve their hydrophobicity.

## Introduction

*Pueraria lobata* (*P. lobata*) is a leguminous plant with important pharmacological activities. The medical effects of *P. lobata* are mostly attributed to the presence of a bioactive ingredient puerarin in its roots As a *C*-glucosylated isoflavone, puerarin exhibits diverse medicinal properties, especially shows the pharmacological activities on cardiovascular and cerebrovascular diseases (Wong et al., 2011; Zhou et al., 2014). Despite of important bioactivities, the low liposolubility and gastrointestinal absorption of puerarin are serious drawbacks for applying it into clinic applications. The 3′-methoxy-puerarin was ever reported from *P. lobata* (Rong et al., 1998; Dubey et al., 2008). Compared to the puerarin itself, its derivative 3′-methoxy-puerarin owns higher liposolubility and antioxidant activity, and better intracellular compartmentation (Heim et al., 2002; Yang et al., 2004; Zhang et al., 2008; Jin et al., 2012). Recent studies showed an obvious protective effect of 3′-methoxy-puerarin on cerebral ischemic-reperfusion injury with some advantages over puerarin (Chen, 2007; Zhang et al., 2008; Han et al., 2009). Considering the better performance of the 3′-methoxy-puerarin, it deserves to identify the *O*-methyltransferase (OMT) in the biosynthetic pathway up to this compound. The methylation at the 3′-OH of the isoflavonoids is likely performed by an *S*-adenosyl-L-methionine (SAM)-dependent OMT, transferring a methyl group from the methyl donor SAM to the hydroxyl group of an acceptor molecule. In plants, OMTs are involved in the methylations of plant secondary metabolites, such as phenylpropanoids, flavonoids, and alkaloids (Ibrahim et al., 1998; Bureau et al., 2007). *O*-methylation of those secondary metabolites could reduce the chemical reactivity of their phenolic hydroxyl groups and increase their lipophilicity or antimicrobial activities (Zhu et al., 1994; Ibrahim et al., 1998; Bureau et al., 2007). From the view of plant physiology, this modifying process plays key roles in lignin biosynthesis, nitrogen fixations and disease resistances (Maxwell et al., 1993; Wu et al., 1997; Boerjan et al., 2003).

**FIGURE 1:**
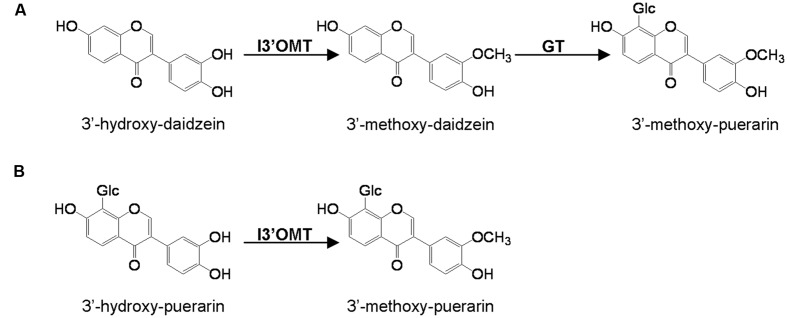
**Proposed two possible pathways (A,B) to 3′-methoxy-puerarin in *Pueraria lobata*.** I3′OMT, isoflavoid 3′-*O*-methyltransferase; GT, glycosyltransferase.

Several cDNA clones encoding isoflavonoid OMTs have recently been isolated and characterized. These include isoflavone 7-OMTs from *Medicago sativa* (He et al., 1998), *M. truncatula* (Deavours et al., 2006), and *Glycyrrhiza echinata* (Akashi et al., 2003) and the 2,7,4′-trihydroxyisoflavanone 4′-OMTs from *M. truncatula* (Deavours et al., 2006), *G. echinata* and *Lotus japonicus* (Akashi et al., 2003). However, to date, the isoflavonoid 3′-OMTs (I3′OMTs) have not been isolated from any plant species and the methylation involved in the biosynthesis of 3′-methoxy-puerarin is not clear. Two possible routes to 3′-methoxy-puerarin are represented in **Figure 1**.

With the goal of isolating the isoflavone 3′-OMT genes, the RNA-sequenced data derived from the leaves and roots of *P. lobata* was extensively analyzed in this study, leading to the identification of two candidate OMTs (designated as PlOMT3 and PlOMT4). Both OMTs were expressed as recombinant proteins and their enzymatic functions were tested. One of the OMTs, PlOMT4, was found to methylate 3′-hydroxy-daidzein to form 3′-methoxy-daidzein. The identification of PlOMT4 clarifies the isoflavone 3′-*O*-methylation process in *P. lobata*, and provides the basis for metabolic engineering of methylated isoflavonoid biosynthesis in other plants and microbial hosts.

## Materials and Methods

### Plant Materials and Chemical

The plant materials of *P. lobata were* collected from the Wuhan botanic garden, Chinese Academy of Sciences. The chemical standard 3′,4′,7-trihydroxyisoflavone (3′-hydroxy-daidzein) was from Toronto Research Chemicals (Toronto, ON, Canada), and the standards prunetin, biochanin A, calycosin, 3′-methoxy daidzein, chrysoeriol, and isoformononetin were purchased from BioBioPha Co., Ltd (Kunming, China). All the other chemicals were purchased from Shanghai Source Leaf Biological Technology Company (Shanghai, China). HPLC grade acetonitrile and methanol (Fisher Scientific) were used for high performance liquid chromatography (HPLC) and liquid chromatography-mass spectrometry (LC–MS). The restriction endonucleases were purchased from Takara Company (Takara, Dalian, China).

### Cloning the Full-Length cDNAs of OMT Candidates

The transcriptome derived from the leaves and roots of *P. lobata* was constructed previously by our group (Wang et al., 2015). BLAST searches against several public databases were conducted in order to collect all the putative OMT sequences. A total of 108 unigenes were annotated as OMTs, of which only 42 unigenes were represented in a length of above one kilo base pairs. Out of these 42 putative OMTs, two candidates were identified as homologs to flavoid-3′-OMTs by BLASTx comparison to the NCBI non-redundant database. The two candidates, designated as PlOMT3 and PlOMT4, were chosen for this study.

The full-length cDNAs of *PlOMT3* and *PlOMT4* were amplified from the roots of *P. lobata* by standard RT-PCRs, and the amplified products were cloned into the pMD18-T vector (Takara) for sequencing confirm. The primers for the PCRs are listed in Supplementary Table S1.

### Phylogenetic Analysis

The deduced amino acid sequences of the OMT candidates from *P. lobata* were aligned with other plant (iso)flavonoid methyltransferases with known functions using the CLUSTAL X Version 2.0 program (Larkin et al., 2007). Then the phylogenetic tree was constructed by the neighbor-joining method of the MEGA 6.0 program (Tamura et al., 2013). The bootstrap value was 1000. The information of the OMTs used in the phylogenetic analysis is summarized in Supplementary Table S2.

### Functional Analysis of OMT Candidates in Yeast

The coding regions of *PlOMT3* and *PlOMT4* were amplified with gene specific primers (Supplementary Table S1) and cloned into the yeast expression vector pESC-HIS (Stratagene, La Jolla, CA, USA) to give the constructs pESC-HIS-PlOMT3 and pESC-HIS-PlOMT4, respectively. Both constructs, as well as the empty vector pESC-HIS as the control, were transferred into the yeast WAT11 strain (Pompon et al., 1996) using the lithium acetate method (Gietz and Woods, 2002). The resulting yeast strains were grown in His dropout liquid medium (Clontech, Palo, CA, USA) containing 2% (w/v) glucose at 30°C. After 24 h, the yeast cells were washed with sterile water and induced by His dropout liquid medium containing 2% (w/v) galactose supplemented with the substrates 3′-hydroxy-daidzein or 3′-hydroxy-puerarin at a final concentration of 100 μM. The yeast cultures were extracted with an equal volume of ethyl acetate after the induction. The ethyl acetate extracts were dried at room temperature and the residue was re-dissolved in 1 ml of methanol for HPLC analysis.

### *In Vitro* Enzyme Assays

The coding region of *PlOMT4* was cloned into the *Escherichia coli* expression vector pET28a (Novagen, Darmstadt, Germany) with its N-terminus fused with a 6 × HIS tag. The primers used for the cloning are listed in Supplementary Table S1. The expression of the recombinant PlOMT4 was induced with 0.5 mM isopropyl-D-thiogalactopyranoside (IPTG) at 16°C for 14 h. The recombinant proteins were purified using HisPur^TM^ Ni-NTA Resin (Thermo) following the provided protocol. The purified proteins were analyzed by SDS-PAGE and protein quantification was determined by Bradford assays.

To check the enzyme activity, enzyme assays were performed in a 200 μl of the reaction mixture consisting of 2 μg of the purified recombinant PlOMT4, 50 mM Tris–HCl (pH 8.0), 2 mM *S*-adenosyl methionine, 2 mM dithiothreitol (DTT) and 100 μM acceptor substrates. The reactions were carried out at 37°C for 20 min, stopped with the addition of 200 μl of methanol, and 15 μl of the reaction mixture was directly used for HPLC analysis.

### Gene Expression and Metabolite Analysis in *P. lobata*

*Porites lobata* materials (roots, leaves, and stems) were collected from open field, powdered under liquid nitrogen, and stored at -80°C for measuring the concentration of 3′-methoxy-daidzein and the transcript abundance of *PlOMT4*. For the methyl jasmonate (MeJA) treatment, a final concentration of 100 μM MeJA was added in the liquid 1/2 MS medium where 1-month-old *P. lobata* seedlings were pre-equilibrated. The same volume of 0.001% ethanol was added into the liquid 1/2 MS medium as the mock control. Roots were collected after 6 h treatment for the gene expression analysis, and after 10 days treatment for the measurement of the concentration of 3′-methoxy-daidzein. To monitor the gene expression in different organs, total RNAs from the roots, stems, and leaves of *P. lobata* were isolated and treated with DNase I (Thermo) to remove genomic DNA contaminations. The first-strand cDNA was synthesized with the SuperScript III reverse-transcriptase (Invitrogen). Quantitative RT-PCR (qRT-PCR) was performed on an ABI 7500 Fast Real-Time PCR Detection System with FastStart Universal SYBR Green Master mix (Rox; Roche, Mannheim, Germany). The PCR conditions were set as follows: 10 min of initial denaturation at 95°C, followed by 40 cycles of 95°C for 15 s and then 60°C for 1 min. All real-time PCRs were performed in three independent repeats. The primers used for the qRT-PCRs are listed in Supplementary Table S1. To measure the concentration of 3′-methoxy daidzein in different organs, 20 mg of plant materials were extracted with 1 ml of ethanol for three times. The ethanol extracts were air dried at room temperature and re-dissolved in 1 ml methanol for HPLC and LC–MS analysis.

### HPLC and LC–MS/MS Analysis

For HPLC analyses, samples were detected by an LC-20AT instrument (Shimadzu, Kyoto, Japan) using an inertsil ODS-SP reverse phase column (250 mm × 4.6 mm, 5 μm) at 30°C. The Milli-Q water containing 0.4% phosphoric acid (solvent A) and HPLC-grade methanol (solvent B) were used as the mobile phase. The samples from the reactions with quercetin, kaempferol, and luteolin were separated using 65% B for 45 min at a flow rate of 0.8 ml/min. For other analyses, 0.1% (v/v) formic acid (A) and acetonitrile (B) were used as the mobile phase and samples were separated as follows: 0–30 min, 25–90% B; 30–35 min, 90–25% B; 35–40 min, 25% B and the flow rate was 0.8 ml min^-1^. The detection wave length was set at 260 nm for isoflavones, 280 nm for liquiritigenin, 350 nm for apigenin and luteolin, and 370 nm for quercetin, kaempferol, and isoliquiritigenin. A calibration standard curve was made from different concentrations (5–100 μg ml^-1^) of each chemical standard. LC–MS/MS analysis was acquired using Accela LC system with TSQ Quantum Access Max mass spectrometer (Thermo Scientific, Waltham, MA, USA) and electrospray ionization source. The analysis method was same as the HPLC analyses described above. The MS data was recorded in a positive ion mode with the ranges of m/z 50–500. Other parameters were set according to those described previously (Li et al., 2014).

## Results

### Isolation and Sequence Analysis of Putative OMT cDNA Candidates

**FIGURE 2:**
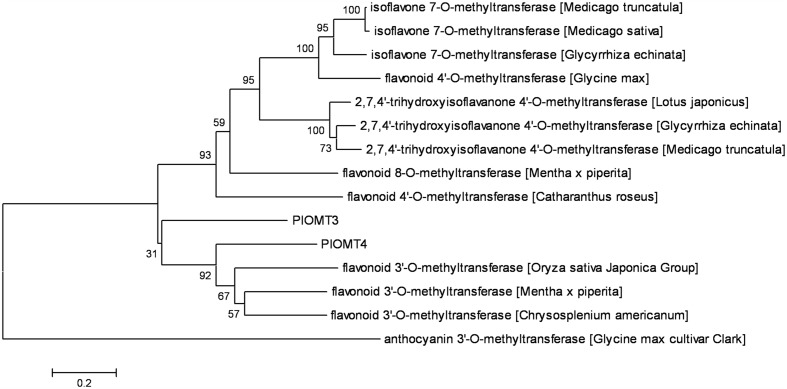
**Phylogenetic analysis of the *P. lobata* OMT candidates with other related sequences.** The tree was constructed by the neighbor-joining method of MEGA 6.0. Numbers on each node indicate the bootstrap values of 1000 replicates. The scale bar represents 0.2 amino acid substitutions per site.

Retrieved from the *P. lobata* transcriptome, the full-length cDNAs of *PlOMT3* and *PlOMT4* were amplified by standard RT-PCRs, and their sequences were confirmed by sequencing and deposited in GenBank with the accession numbers of KP057886 and KP057887, respectively. The deduced amino acid sequences of the two OMT candidates showed 37.8% identity with each other. A phylogenetic tree was constructed to examine the relationships of the OMT candidates with other (iso)flavoid OMTs from other plant species whose functions are already known (**Figure 2**). PlOMT3 and PlOMT4 clustered with the flavonoid 3′-OMTs, suggesting that they are the candidates for the isoflavone 3′-*O*-methylation in *P. lobata*.

### Functional Analysis of the OMT Candidates in Yeast

**FIGURE 3:**
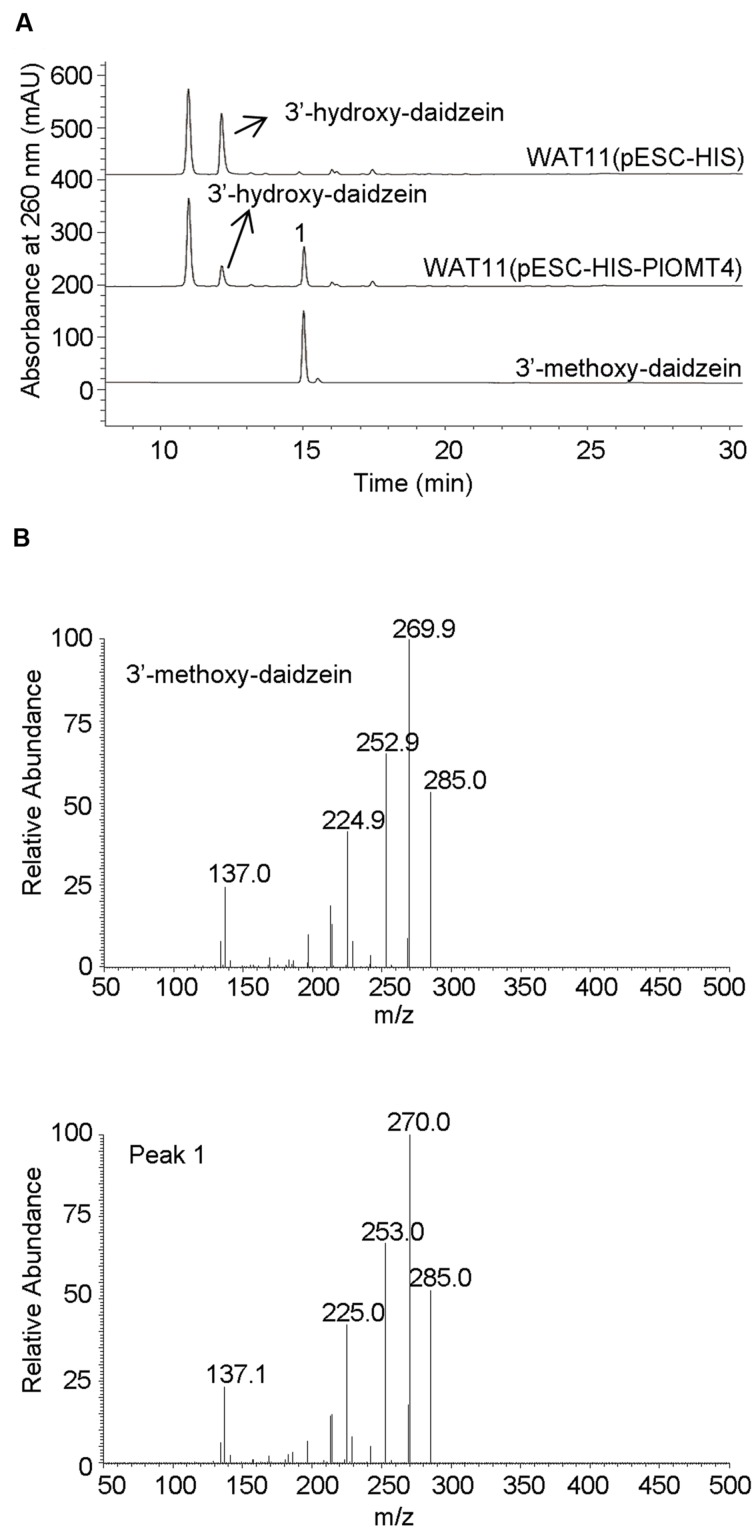
**Biochemical characterization of PlOMT4 in yeast cells by LC–MS analysis. (A)**, the conversion of the substrate 3′-hydroxy-daidzein to 3′-methoxy-daidzein (peak 1) by PlOMT4; **(B)**, the mass spectra of the peak 1 with its authentic standard. WAT11 (pESC-HIS), the yeast strain carrying the empty vector pESC-HIS as a control; WAT11 (pESC-HIS-PlOMT4), the yeast strain expressing PlOMT4.

To screen the activities of PlOMT3 and PlOMT4, the two possible substrates 3′,4′,7-trihydroxyisoflavone (3′-hydroxy-daidzein**)** and 3′-hydroxy-puerarin, as shown in **Figure 1**, were added to the yeast cultures that expressed the recombinant PlOMTs. After the induction, the yeast cultures were extracted with ethyl acetate and the conversion of the substrates were examined by HPLC analysis. Notably, yeast cells expressing PlOMT4 could convert the substrate 3′-hydroxy-daidzein to form the 3′-methoxy-daidzein (**Figure 3A**), indicating the 3′-*O*-methylation activity of PlOMT4. When the 3′-hydroxy-puerarin was added to the PlOMT4-expressed culture, no apparent conversion of the substrate was observed, in comparison with the control culture harboring the empty vector (data not shown). This data demonstrated that PlOMT4 is able to methylate the 3′-hydroxy-daidzein but not accept the 3′-hydroxy-puerarin as the substrate. On the other hand, both the 3′-hydroxy-daidzein and 3′-hydroxy-puerarin were not significantly converted by the PlOMT3-expressed cultures (data not shown), suggesting that PlOMT3 exhibited no activities toward these two substrates. The identity of the product 3′-methoxy-daidzein was confirmed with its standard using LC–MS analysis (**Figure 3B**).

### *In Vitro* Enzyme Assays

**FIGURE 4:**
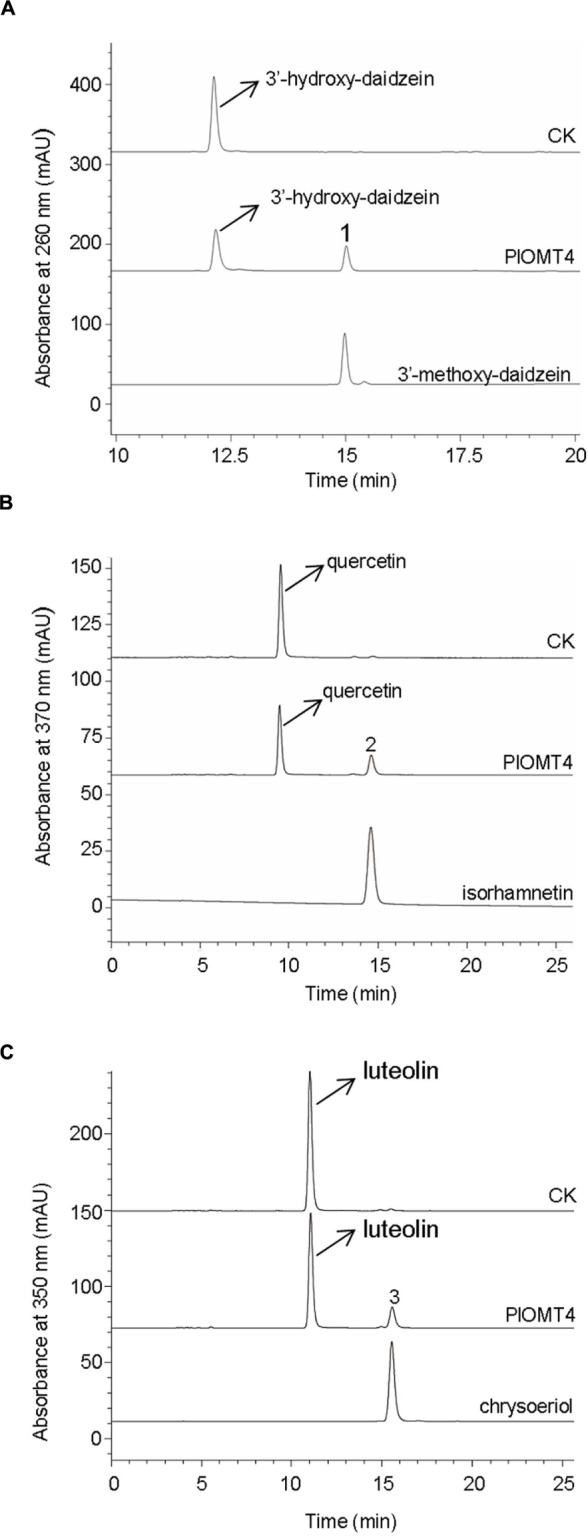
**LC–MS analyses of the enzyme activities of the purified recombinant PlOMT4 *in vitro.*** The enzyme activities of the recombinant PlOMT4 were shown for the conversion of 3′-hydroxy-daidzein to 3′-methoxy-daidzein (peak 1; **A**), the conversion of quercetin to isorhamnetin (peak 2; **B**), and the conversion of luteolin to chrysoeriol (peak 3; **C**). The mass spectra of the peaks 1–3 with their corresponding authentic standards were shown in Supplementary Figure S3.

To examine the substrate specificity of PlOMT4, PlOMT4 was successfully expressed in *E. coli* BL21 (DE3) cells and purified using HisPur^TM^ Ni-NTA Resin (Thermo). The molecular weight of the recombinant PlOMT4 was approximately 45 kDa, which is consistent with its predicted molecular weight (Supplementary Figure S1), suggesting an integrity of the PlOMT4 protein. The purified recombinant PlOMT4 were then assayed *in vitro* with a broad range of substrates (Supplementary Figure S2), including one phenolic acid (caffeic acid), one chalcone (isoliquiritigenin), one flavanone (liquiritigenin), two flavones (apigenin and luteolin), two flavonols (quercetin and kaempferol), and eight isoflavones (daidzein, genistein, formononetin, biochanin A, prunetin, isoformonetin, 3′-hydroxy-daidzein, and 3′-hydroxy-puerarin). Consistent with the *in vivo* assays described above, the purified PlOMT4 methylated the substrate 3′-hydroxy-daidzein to form the product 3′-methoxy-daidzein (peak 1) (**Figure 4A**). In addition, the substrates quercetin and luteolin were also converted by PlOMT4, producing their respective methylated products isorhamnetin (peak 2) and chrysoeriol (peak 3), respectively (**Figures 4B,C**). The identities of the products (peaks 1–3) were confirmed with their chemical standards by LC–MS analysis (Supplementary Figure S3). In contrast, none of the other substrates used were converted by the recombinant PlOMT4 (data not shown), especially, the 3′-hydroxy-puerarin was not converted, suggesting that the methylation must occur prior to the glycosylation of the isoflavone backbone.

The initial velocities of PlOMT4 with 3′-hydroxy-daidzein, luteolin, and quercetin were then compared. As shown in **Table 1**, the purified PlOMT4 exhibited the highest activity toward the isoflavone 3′-hydroxy-daidzein (100% relative activity, 4180.4 pkat/mg) with a relatively lower activity with the flavone luteolin (42.1% relative activity, 1759.5 pkat/mg) and the flavonol quercetin (27.3% relative activity, 1142.3 pkat/mg).

**Table 1:** Enzymatic activities of the purified recombinant PlOMT4 toward a range of substrates *in vitro*.

Substrate	Enzyme activity (pkat/mg)	Relative activity (%)^a^
**Phenolic acid**		
Caffeic acid	0	0
**Isoflavone**		
3′-Hydroxy-daidzein	4180.4 ± 150.5	100 ± 3.6
Daidzein	0	0
Genistein	0	0
Formononetin	0	0
Biochanin A	0	0
Prunetin	0	0
Isoformononetin	0	0
**Chalcone**		
Isoliquiritigenin	0	0
**Flavone**		
Apigenin	0	0
Luteolin	1759.9 ± 142.1	42.1 ± 3.4
**Flavanone**		
Liquiritigenin	0	0
**Flavonol**		
Quercetin	1142.3 ± 54.4	27.3 ± 1.3
Kaempferol	0	0

^a^*Relative activity. The values are normalized with the most converted substrate 3′-hydroxy-daidzein as 100%.*

### The Relevance of PlOMT4 Transcripts and Its Catalyzed Metabolites in *P. lobata*

**FIGURE 5:**
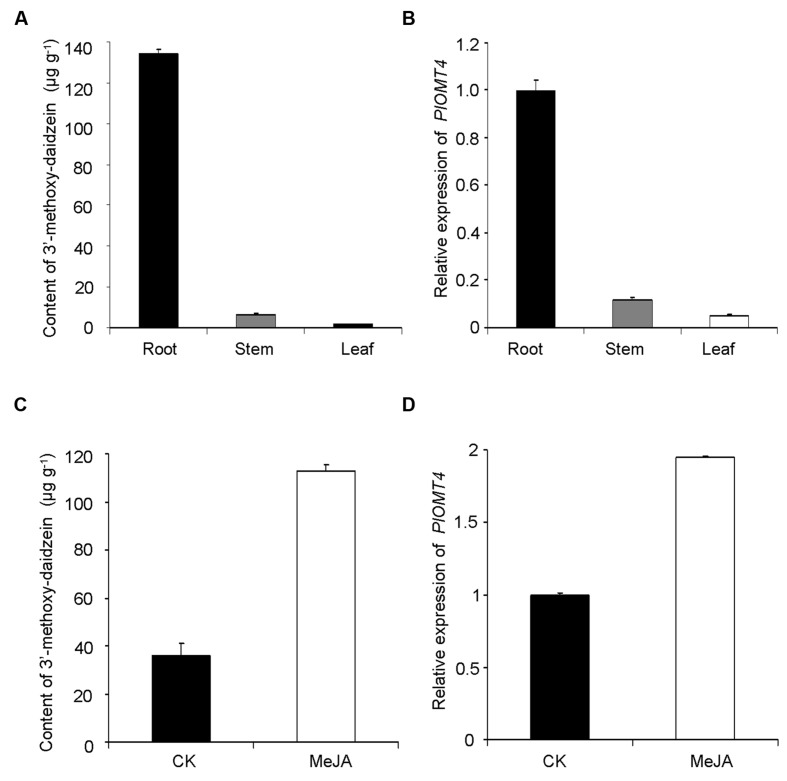
**Correlation analysis of the *PlOMT4* transcript abundance with the accumulation of the 3′-methoxy-daidzein. (A)**, the accumulation pattern of 3′-methoxy-daidzein in the different organs of *P. lobata*; **(B)**, the relative abundance of the *PlOMT4* transcript in the different organs; **(C)**, the increment of the accumulation of the 3′-methoxy-daidzein in the *P. lobata* roots in response to the MeJA stimuli; **(D)**, the elevation of the *PlOMT4* transcripts in the *P. lobata* roots by the MeJA treatment. The *P. lobata* actin gene (GenBank accession no. HO708075) was used as an internal standard for the gene expression analysis.

The enzymatic product of PlOMT4, 3′-methoxy-daidzein, was mostly detected in the roots (133.9 ± 2.7 μg g^-1^ dry weight), but was almost undetectable in its stems or leaves (**Figure 5A**). Consistent with the metabolite accumulation, the highest abundance of the *PlOMT4* transcripts was observed in the roots whereas extremely low expressions were found in the stems and leaves (**Figure 5B**). Thus, the *PlOMT4* transcripts match well with the accumulation pattern of its enzymatic product 3′-methoxy-daidzein in *P. lobata* in a spatial manner.

To further examine the relevance, the gene transcripts and the metabolite accumulation were monitored in an eliciting manner, by treating the 1-month-old *P. lobata* seedlings with 100 μM MeJA. The roots of 10 days-treated plants were used for metabolite analysis while 6 h-treated plants were collected for measuring the gene transcript levels. Apparently, the MeJA stimuli significantly increased the biosynthesis of the product 3′-methoxy-daidzein in comparison with the mock control roots (**Figure 5C**). In line with the increase of the metabolite accumulation, the transcript concentration of *PlOMT4* was also elevated in response to the MeJA treatment (**Figure 5D**). Taken together, The *PlOMT4* transcript abundance correlates well with the accumulation of 3′-methoxy-daidzein in *P. lobata*, supporting its proposed biochemical role *in vivo*.

## Discussion

Many isoflavonoids are *O*-methylated with the methoxy residue enhancing their biological activities by altering hydrophobicity. The common sites for the methylation are the 3′-, 4′- and 7-positions of isoflavonoids (isoflavone carbon numbering). Either the 4′- or 7-methylation activity has been studied in several legume species (He et al., 1998; Akashi et al., 2003; Deavours et al., 2006), however, there was no report on the identification of a gene that encodes the isoflavone 3′-OMT (I3′OMT). Phytochemical work has elucidated the presence of 3′-*O*-methylated isoflavones in *P. lobata*, which allows it to be a suitable system for the molecular investigation of the isoflavone 3′-*O*-methylation. In this study, we isolated the first cDNA coding for an I3′OMT (PlOMT4) from *P. lobata*. Heterologous expression in yeast and *E. coli* showed that the gene product exhibited an enzyme activity to methylate the isoflavone substrate 3′-hydroxy-daidzein at the 3′ position. The closest homolog of PlOMT4 in sequence databases (44.9% identity) is an enzyme with the flavonoid 3′-OMT (F3′OMT) activity from *Mentha* x *piperita* (Willits et al., 2004), which probably indicates that PlOMT4 might diverge from F3′OMTs. PlOMT4 preferentially methylates 3′-hydroxy-daidzein, it does have some but relatively lower activities on flavonoid compounds with 3′-hydroxy moiety, e.g., luteolin and quercetin (**Figure 4**). In particular, no detectable products resulted when the glycosyl derivatives of 3′-hydroxy-daidzein, i.e., 3′-hydroxy puerarin, were incubated with PlOMT4, suggesting that the isoflavone modifying reactions behave in strict orders and the 3′-*O*-methylation may proceed prior to glycosylations *in vivo*. The biochemical function of PlOMT4 allowed us to propose the route A shown in **Figure 1** as the favorable pathway to 3′-methoxy-puerarin. By reference to the structural basis for plant isoflavone OMTs (Zubieta et al., 2001), the residues His-277, Asp-305, and Glu-337 of PlOMT4 might be important for its biochemical functions.

The gene expression of *PlOMT4* in relation to the 3′-*O*-methylated isoflavonoid profile of *P. lobata* was studied. 3′-methoxy-daidzein are almost exclusively accumulated in *P. lobata* roots (**Figure 5A**), which is consistent with the distribution pattern of the *PlOMT4* transcripts (**Figure 5B**). Moreover, in the *P. lobata* roots, the gene expression levels of *PlOMT4* were increased by MeJA elicitation (**Figure 5C**), which also resulted in the increment of the 3′-*O*-methylated isoflavonoid biosynthesis (**Figure 5D**), suggesting that methylated isoflavonoids function as phytoalexins (Ibrahim et al., 1998; Bureau et al., 2007). The integration of the enzyme biochemical property *in vitro*, gene expression and metabolite accumulation *in vivo* strongly supported the proposed role of PlOMT4 in *P. lobata*.

## Conclusion

PlOMT4 is the first I3′OMT identified from plants and it is likely responsible for the *O*-methylation of *P. lobata* isoflavones at C-3′ position. The identification of PlOMT4 should provide an opportunity to improve lipophilicity of isoflavonoids or other structure-like compounds by *O*-methylations at appropriate positions and in turn push forward for clinical applications.

## Author Contributions

YZ designed this study; JL performed the gene cloning and biochemical reactions; CL performed the MeJA treatment and provided the assistance in LC–MS or HPLC analysis; JG helped to collect the plant materials; JL and YZ wrote the manuscript.

## Conflict of Interest Statement

The authors declare that the research was conducted in the absence of any commercial or financial relationships that could be construed as a potential conflict of interest.
