# Supplementary material for: Molecular Cloning and Functional Characterization of a Novel Isoflavone 3′-O-methyltransferase from Pueraria lobata
Source: Front Plant Sci. 2016 Jun 6;7:793. doi: 10.3389/fpls.2016.00793 (PMC4937802; doi:10.3389/fpls.2016.00793)
Supplement: Supplementary file 1 [file Data_Sheet_1.DOC]

Supplementary Material

Molecular cloning and functional characterization of a novel isoflavone 3′-*O-*methyltransferase from *Pueraria lobata*

Jia Li, Changfu Li, Junbo Gou, Yansheng Zhang*****

***Correspondence:** Yansheng Zhang: [zhangys@wbgcas.cn](mailto:zhangys@wbgcas.cn)

# Supplementary Figures and Tables

## Supplementary Figures

**
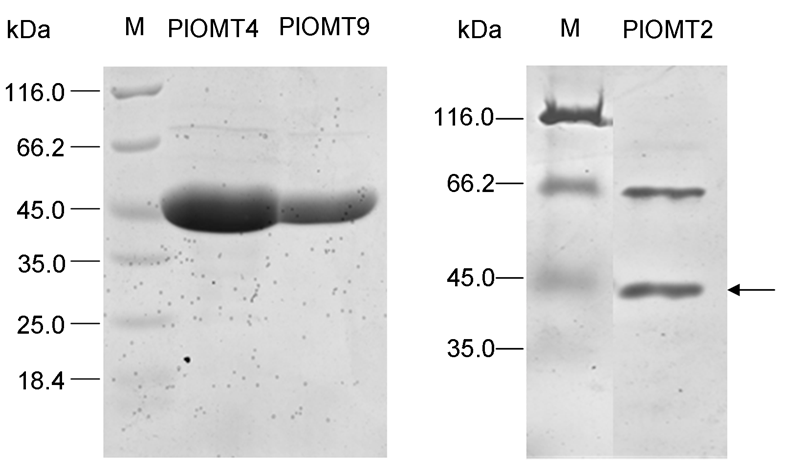
**

**Supplementary Figure 1.** SDS-PAGE electrophoresis analysis of the purified recombinant PlOMT4*.*

**
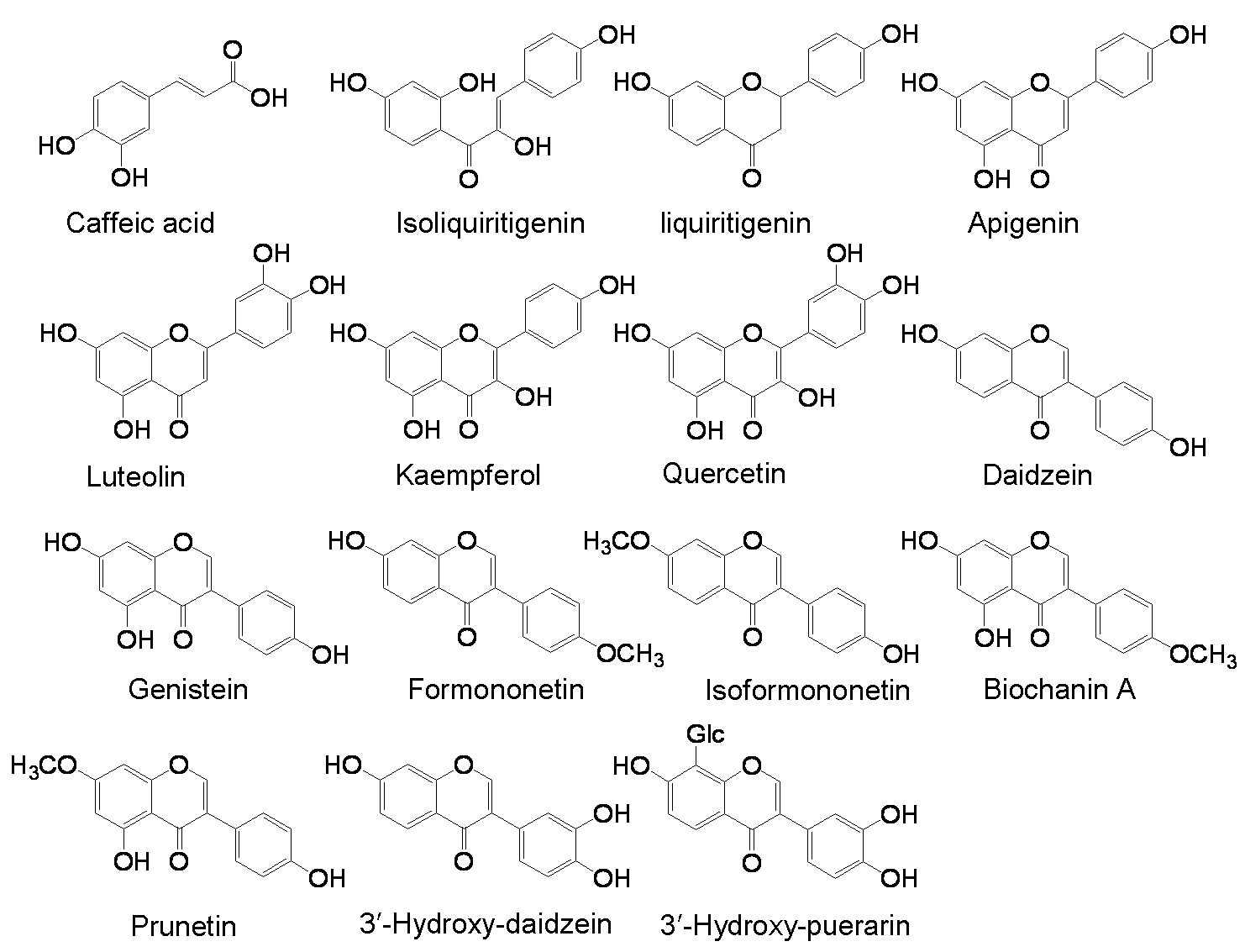
**

**Supplementary Figure 2.** Structure of all the substrates used for the enzyme assays of PlOMT4 *in vitro*.

**
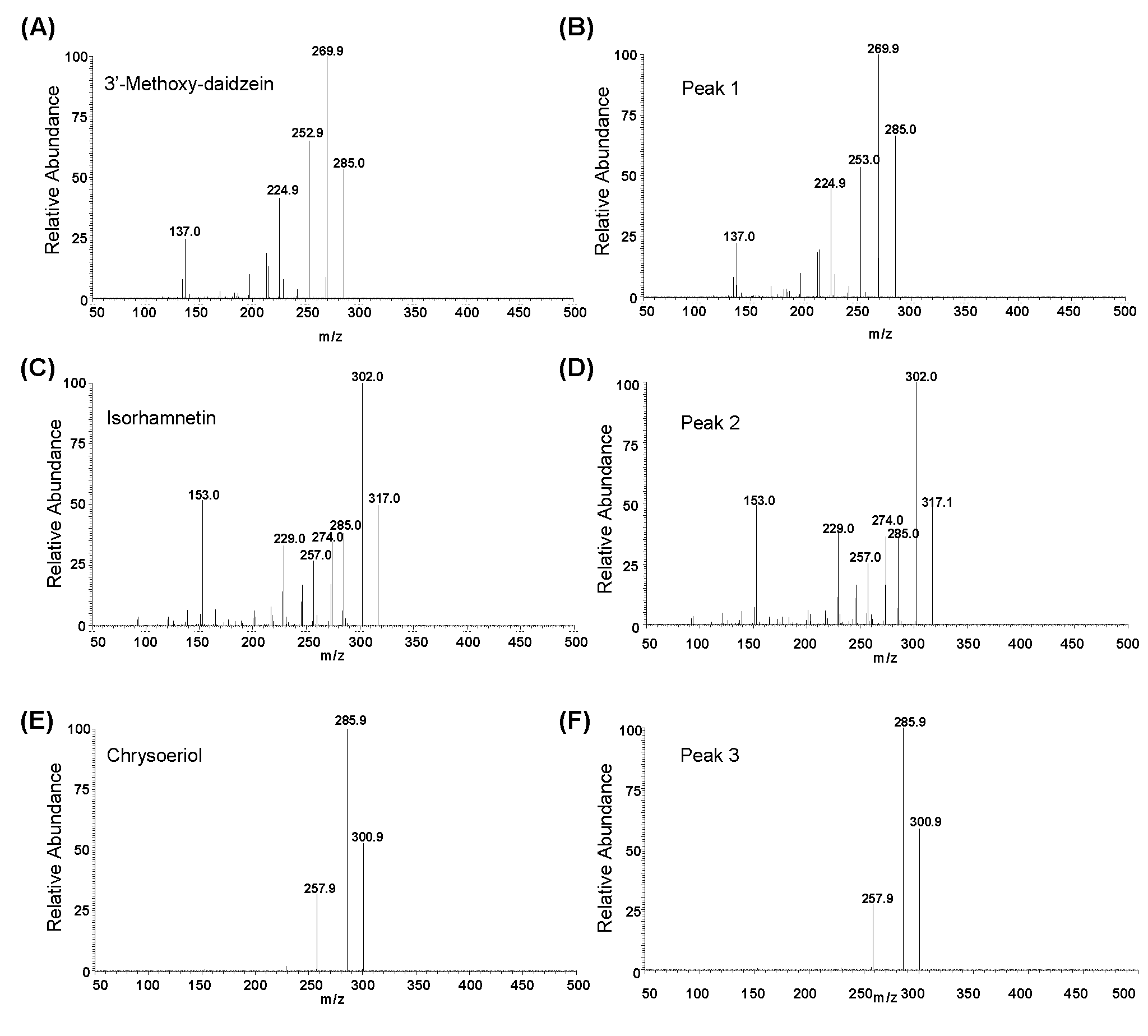
**

**Supplementary Figure 3.** Mass spectra of the products (peaks 1-3) shown in Figure 4 from the main file**.** The mass spectrums were shown for the authentic 3′-methoxy-daidzein (A), the 3′-methoxy-daidzein product (peak 1) (B), the authentic isorhamnetin (C), the isorhamnetin product (peak 2) (D), the authentic chrysoeriol (E), and the chrysoeriol product (peak 3) (F). The collision energy used for LC-MS/MS analysis of the 3′-methoxy-daidzein or chrysoeriol is 25 V, and that of isorhamnetin is 30 V.

## Supplementary Tables

**Supplementary Table 1.** Primers used for the gene amplifications in this study

| Genes | Primers 5' to 3' sequence |
| --- | --- |
| For pMD18-T /pESC-His |  |
| *PlOMT3* (upstream) | CCCATCGATGGAGACTGTTCTTTTCAATCAC |
| (downstream) | CCCGAGCTCTCAGATTGGAAATGCCTCAAT |
| *PlOMT4*  (upstream) | CCCAGATCTATGGCTCCATCATTGGAAAC |
| (downstream) | CCCGTCGACCTACTTATAAAACTCCATAACCC |
| For pET28a |  |
| *PlOMT4*  (upstream) | CCCAGATCTATGGCTCCATCATTGGAAAC |
| (downstream) | CCCGTCGACCTACTTATAAAACTCCATAACCC |
| For gene expression analyses |  |
| *Actin* (upstream) | GGGGTGATGGTTGGGATG |
| (downstream) | CGGTGAGAAGAACAGGGT |
| *PlOMT4*  (upstream) | CCGTGTTCTCAGGTTATTGTC |
| (downstream) | CTCCTTTCAGTTCGGTCCAG |

**Supplementary Table 2.** Information of the methyltransferases used in the phylogenetic analysis

| GenBank accession Number | Plant | Function |
| --- | --- | --- |
| AAY18582 | *Medicago truncatula* | isoflavone 7-*O*-methyltransferase |
| AAC49927 | *M. sativa* | isoflavone 7-*O*-methyltransferase |
| BAC58012 | *Glycyrrhiza echinata* | isoflavone 7-*O*-methyltransferase |
| BAC58011 | *G. echinata* | 2,7,4′-trihydroxyisoflavanone 4'-*O*-methyltransferase |
| BAC58013 | *Lotus japonicus* | 2,7,4′-trihydroxyisoflavanone 4'-*O*-methyltransferase |
| AAY18581 | *M. truncatula* | 2,7,4′-trihydroxyisoflavanone 4'-*O*-methyltransferase |
| AAR02420 | *Catharanthus roseus* | flavonoid 4′-*O*-methyltransferase |
| C6TAY1 | Glycine *max* | flavonoid 4′-*O*-methyltransferase |
| AAR09601 | *Mentha* x *piperita* | flavonoid 3′-*O*-methyltransferase |
| ABB90678 | *Oryza sativa* | flavonoid 3′-*O*-methyltransferase |
| AAA80579 | *Chrysosplenium americanum* | flavonoid 3′-*O*-methyltransferase |
| ADX43927 | G. *max* | anthocyanin 3′-*O*-methyltransferase |
| AAR09600 | *Mentha* x *piperita* | flavonoid 8*-O-*methyltransferase |
